# Potential for a Virtual Care Model in the Perioperative Management of Anticoagulant Therapy: A 5-Year Retrospective Clinic Review

**DOI:** 10.1055/a-2098-6782

**Published:** 2023-07-05

**Authors:** James Luke Douketis, Sam Schulman

**Affiliations:** 1 Department of Medicine, Thrombosis and Atherosclerosis Research Institute, McMaster University, Hamilton, Ontario, Canada; 2 Department of Obstetrics and Gynecology, I.M. Sechenov First Moscow State Medical University, Moscow, Russia

**Keywords:** virtual care, perioperative, anticoagulant, heparin bridging, warfarin, direct oral anticoagulants

## Abstract

**Background** With a trend toward greater virtual care in selected clinical settings, perioperative anticoagulant management appears well suited for this care delivery model. We explored the potential for virtual care among patients who are receiving anticoagulant therapy and require perioperative management around the time of an elective surgery/procedure.

**Methods** We undertook a retrospective review of patients who were receiving anticoagulant therapy, either a direct oral anticoagulant (DOAC) or warfarin, assessed in a perioperative anticoagulation-bridging clinic over a 5-year period from 2016 to 2020. Using prespecified criteria, we determined the proportion of patients who likely would be suitable for virtual care (receiving a DOAC or warfarin and having a minimal- or low-/moderate-bleed-risk surgery/procedure), those who likely would be suitable for in-person care (receiving warfarin and requiring heparin bridging for a mechanical heart valve), and patients who would be suitable for either care delivery model (receiving a DOAC or warfarin, but not with a mechanical heart valve, and requiring a high-bleed-risk surgery/procedure).

**Results** During the 5-year study period, there were 4,609 patients assessed for perioperative anticoagulant management in whom the most widely used anticoagulants were warfarin (37%), apixaban (30%), and rivaroxaban (24%). Within each year assessed, 4 to 20% of all patients were undergoing a minimal-bleed-risk procedure, 76 to 82% were undergoing a low-/moderate-bleed-risk surgery/procedure, and 10 to 39% were undergoing a high-bleed-risk surgery/procedure. The proportion of patients considered suitable for virtual, in-person, or either virtual or in-person management was 79.6, 7.1, and 13.3%, respectively.

**Conclusion** In patients who were assessed in a perioperative anticoagulation clinic, there was a high proportion of patients in whom a virtual care model might be suitable.

## Introduction

The perioperative management of patients who are receiving anticoagulant therapy and require a surgery/procedure is a common clinical problem, with approximately 4 million patients assessed in North America each year. 1 2 These patients, typically, are receiving a direct oral anticoagulant (DOAC), which comprises oral factor Xa inhibitors (apixaban, edoxaban, rivaroxaban) and an oral factor II inhibitor (dabigatran), 3 or a vitamin K antagonist (VKA), which is usually warfarin. To assess and manage such patients, standardized perioperative anticoagulant protocols have been developed based on high-quality randomized controlled trials and prospective observational management studies. 4 5 6 7 8 9 However, little attention has been dedicated to the delivery of perioperative anticoagulant management and the infrastructure needed to capably deliver such patient care. 10 Most importantly, the COVID-19 pandemic has upended the traditional, in-person, model of care delivery with a shift toward virtual care in several clinical domains, including perioperative management. 11 12 13

It is possible that a substantial proportion of future perioperative anticoagulant management can be delivered virtually, as this care delivery model is well suited to perioperative management because standardized, evidence-based perioperative management protocols are available through user-friendly, point-of-care, web-based resources ( www.thrombosiscanada.ca ; http://mappp.ipro.org ; http://www.maqi2.org ) that allow input of patient-specific clinical information with an output of a perioperative anticoagulant care path that can be shared with patients, families, and the multidisciplinary health care team. 10 14

In anticipation of a potential shift from an in-person to a virtual model of care delivery for perioperative anticoagulant management, the objective of this study is to describe the characteristics of patients attending a perioperative anticoagulation-bridging clinic and to ascertain which patients would be eligible to receive virtual management, in-person management, or either model of care delivery based on their anticipated needs. Specifically, we aimed to determine the proportion of patients that can be classified in one of three groups: (1) those likely to be safely managed virtually, defined as patients having a low-bleed-risk surgery/procedure with anticoagulation (DOAC, warfarin) interruption but without heparin bridging or a minimal-bleed-risk procedure where no anticoagulant interruption is needed; (2) those likely to require in-person management, defined as patients who are receiving warfarin and likely require perioperative heparin bridging; and (3) those who might be managed with *either* an in-person or virtual approach, defined as patients receiving an anticoagulant (DOAC, warfarin) and are undergoing a high-bleed-risk surgery.

## Methods

### Study Design and Patients

We performed a descriptive, retrospective medical record review (without patient contact) to gather information on consecutive patients who were assessed in the perioperative anticoagulation (bridging) clinic at the Hamilton General Hospital over a 5-year period, from January 1, 2016 to December 31, 2020. The study period chosen was based on the year during which the data extraction was performed (2021) coupled with the aim of including a 5-year study period. We anticipated that the volume of data from the first pandemic year (2020) would be less than in previous years due to pandemic-related disruptions in care delivery leading to cancellations or delays in elective surgery/procedures. A retrospective study design is appropriate to address questions related to a description of patient characteristics attending a clinic.

The study was approved by the Hamilton Integrated Research Ethics Board. There was no funding provided for this study.

### Clinical Setting

The perioperative anticoagulation management clinic is situated in a multidisciplinary outpatient department. The clinic takes place 4 days per week, with 8 to 10 patients assessed daily who are receiving warfarin or a DOAC. A nurse practitioner assesses and manages patients using standardized protocols, with support and direction from a consultant physician. 10 Additional staff include an administrative assistant, who manages referrals and bookings. Patients are referred, typically, 10 to 14 days in advance of the planned surgery/procedure, but the clinic also accepts last minute and urgent cases. The clinic encourages participation of medical trainees from medical, surgical, and anesthesia departments.

### Data Collection

Data collection was organized in a password-protected Excel workbook. Names of patients, along with clinical indication for anticoagulation, surgery/procedure type, date of surgery/procedure, and patient number, were obtained from a preexisting database. We captured information from the patient records of the clinic comprising the following: (1) patient number; (2) year of surgery/procedure; (3) anticoagulant type, comprising a DOAC or warfarin; (4) clinical indication for anticoagulation, comprising atrial fibrillation/flutter (AF), mechanical heart valve (MHV), venous thromboembolism (VTE), or other clinical indication (i.e., including cerebrovascular disease, cardiomyopathy, peripheral arterial disease); and (5) surgery-/procedure-related bleed risk, classified as high-bleed-risk, low-/moderate-bleed-risk, or minimal-bleed-risk. The bleed risk classification used was based on a prespecified classification scheme shown in Appendix 1 . Data pertaining to patient risk for thromboembolism, for example, to calculate a CHADS _2_ VA _2_ Sc score in patients with AF or to determine risk of disease recurrence in patients with VTE, were incomplete; consequently, we were unable to reliably determine thromboembolic risk and did not include this in the description of patient characteristics.

**Appendix 1:** Surgery/procedure-related bleeding risk classification

High-bleed-risk surgery/procedure (30-d risk of major bleed ≥2%)	● Major surgery with extensive tissue injury ● Cancer surgery, especially solid tumor resection (lung, esophagus, gastric, colon, hepatobiliary, pancreatic) ● Major orthopaedic surgery, including shoulder replacement surgery ● Reconstructive plastic surgery ● Major thoracic surgery ● Urologic or gastrointestinal surgery, especially anastomosis surgery ● Transurethral prostate resection, bladder resection, or tumor ablation ● Nephrectomy, kidney biopsy ● Colonic polyp resection ● Bowel resection ● Percutaneous endoscopic gastrotomy (PEG) placement, endoscopic ● Retrograde cholangiopancreatography (ERCP) ● Surgery in highly vascular organs (kidneys, liver, spleen) ● Cardiac, intracranial, or spinal surgery ● Any major operation (procedure duration >45 min) ● Neuraxial anesthesia a ● Epidural injections
Low-/moderate-bleed-risk surgery/procedure (30-d risk of major bleed 0-2%)	● Arthroscopy ● Cutaneous/lymph node biopsies ● Foot/hand surgery ● Coronary angiography b ● Gastrointestinal endoscopy░±░biopsy ● Colonoscopy░±░biopsy ● Abdominal hysterectomy ● Laparoscopic cholecystectomy ● Abdominal hernia repair ● Hemorrhoidal surgery ● Bronchoscopy░±░biopsy
Minimal-bleed-risk surgery/procedure (30-d risk of major bleed ~0%)	● Minor dermatologic procedures (excision of basal and squamous cell skin cancers, actinic keratoses, and premalignant or cancerous skin nevi) ● Ophthalmological (cataract) procedures ● Minor dental procedures (dental extractions, restorations, prosthetics, endodontics), dental cleanings, and fillings ● Pacemaker or cardioverter-defibrillator device implantation

a Includes spinal and epidural anesthesia or any other neuraxial (e.g., pain management) intervention; consider not only absolute risk for major bleeding but also potentially devastating consequences of epidural bleeding and associated lower limb paralysis.

b Radial approach may be considered minimal bleed risk compared to the femoral approach.

### Data Extraction

To obtain the requisite patient information, digital databases were examined initially followed by paper files if digital-based data were not used. The anticoagulant used was obtained from either patient information forms or weekly medication charts. If information was not collected from previously mentioned places, physicians' clinic notes were examined.

### Data Capture and Transfer

To maintain patient confidentiality for online databases, patient number was used as the main identifier of the patient. Since physical files were in the alphabetical order, the patient's name and patient number were used as co-identifiers of the patient. For patients whose physical files could not be found, electronic databases on hard drives were consulted. Data were extracted on a yearly basis by search functions and put into charts. These charts were then graphed. Patients were excluded if their files could not be found; this comprised 320 patients. In addition, 130 patients were excluded who were assessed in the perioperative anticoagulation clinic but in whom their surgery/procedure was canceled.

### Statistical Analysis

We determined the proportion of patients who would be candidates for virtual perioperative anticoagulant management. We also determined the proportion of patients who would be candidates for in-person perioperative anticoagulant management, specifically if they were receiving warfarin and required heparin bridging because of an MHV. We did not consider other patient groups who were taking warfarin with AF or VTE to require heparin bridging as our practice is to rarely bridge such patients, Finally, we determined the proportion of patients who would be eligible for virtual or in-person management.

## Results

### Patient Population

Over a 5-year period of study from January 1, 2016 to December 31, 2020, there were a total of 4,609 patients assessed in the perioperative bridging anticoagulation clinic, with 1,083, 861, 1,001, 1,009, and 655 patients assessed in 2016, 2017, 2018, 2019, and 2020, respectively; on average, 922 patients were assessed each year. The volume of patients assessed in 2020 was reduced because of pandemic-related delays or cancellations of elective surgeries and procedures.

Table 1 shows the type of anticoagulant patients were receiving according to the clinical indication, and Table 2 shows the anticoagulant type patients were receiving by year from 2016 to 2020. Overall, the most widely used anticoagulant was warfarin (37%), followed by apixaban (30%) and rivaroxaban (24%); 63% of patients were receiving a DOAC. Over the 5-year study period, the number of warfarin users assessed appeared to decline, whereas the number of DOAC users, especially those taking apixaban or rivaroxaban, appeared to increase.

**Table 1:** Clinical Indication for anticoagulant therapy

	Clinical indication for anticoagulant therapy				
Anticoagulant type	VTE	MHV	AF	Other	Total (%)
Apixaban	134	0	1,162	87	1,383 (30)
Dabigatran	15	0	324	25	364 (8)
Edoxaban	11	0	44	3	58 (1)
Rivaroxaban	240	0	857	97	1,094 (24)
Warfarin	311	385	788	126	1,710 (37)
Total (%)	711 (15)	385 (8)	3,175 (70)	338 (7)	4,609

Abbreviations: AF, atrial fibrillation; MHV, mechanical heart valve; MVR, mechanical heart valve; VTE, venous thromboembolism.

**Table 2:** Anticoagulant type managed, by year

Anticoagulant type	2016	2017	2018	2019	2020	Total (%)
Apixaban	264	252	345	328	194	1,383 (30)
Dabigatran	132	71	62	68	31	364 (8)
Edoxaban a	0	0	21	18	19	58 (1)
Rivaroxaban	228	215	255	246	150	1,094 (24)
Warfarin	459	323	318	349	261	1,710 (37)
Total (%)	1,083 (23)	861 (19)	1,001 (22)	1,009 (22)	655 (14)	4,609

a Not approved for clinical use in Ontario, Canada until 2018.

### Bleed-Risk Category of Surgery/Procedures

Over the 5-year study period, 4 to 10% of patients assessed were classified as undergoing a minimal-bleed-risk surgery/procedure, 67 to 86% of patients were classified as low-/moderate-bleed-risk surgery/procedure, and 10 to 39% were classified as high-bleed-risk surgery/procedure. It is noteworthy that the proportion of patients assessed who were classified as undergoing a high-bleed-risk surgery/procedure appeared to decline over the study period (39, 19, 10, 14, and 10%).

In terms of bleed-risk category according to the clinical indication for anticoagulant therapy, shown in Table 3 , the low-/moderate-bleed-risk group was dominant, irrespective of the indication for anticoagulant therapy, comprising 76 to 82% of all patients assessed. There were few patients having a minimal-bleed-risk surgery/procedure, comprising 4 to 20% of patients assessed across clinical indications.

**Table 3:** Potential for virtual, in-person or either model of patient management

	Clinical indication for anticoagulant therapy				
Bleed risk category	VTE	AF	MHV	Other	Total (%)
Minimal	60	142	57	23	282 (6.2)
Low/moderate	521	2,603	200	293	3,617 (78.6)
High	130	430	128	52	740 (16.2)

Abbreviations: AF, atrial fibrillation; MHV, mechanical heart valve; VTE, venous thromboembolism.

Note: Lightly shaded cells represent patients who might be assessed virtually (80%); darkly shaded cells represent patients that likely require in-person assessment (7%); medium-shaded cells represent patients who might be assessed in person or virtually (13%).

### Potential for Virtual or In-person Perioperative Anticoagulant Management

#### Virtual Management Likely Adequate

As shown in Table 3 (lightly shaded cells), this group comprised DOAC and warfarin users undergoing a minimal-bleed-risk surgery/procedure, in whom anticoagulant interruption was not needed, and DOAC and warfarin users with VTE or AF who were undergoing a low-/moderate-bleed-risk surgery/procedure and in whom heparin bridging would not be warranted. There were 6.1% patients who were taking a DOAC or warfarin and were in the minimal-bleed-risk procedure category and 73.5% DOAC-treated patients in the low-/moderate-bleed-risk category. When these two groups were combined, this represented 79.6% (3,669) of all patients assessed in whom virtual perioperative management might be adequate.

#### In-Person Management Likely Needed

This group comprised warfarin-treated patients with an MHV who were undergoing a low-/moderate- or high-bleed-risk surgery/procedure in whom warfarin interruption would be warranted and heparin bridging likely would be needed. Overall, there were 7.1% (328) patients in this category.

#### Virtual or In-Person Management Likely Adequate

The remaining patients falling into this category were DOAC-treated patients undergoing a high-bleed-risk surgery/procedure, which comprised 13.3% (612) patients. We were unable to reliably identify warfarin-treated patients with AF or VTE undergoing a low-/moderate-bleed-risk surgery/procedure who were considered at high risk of thromboembolism (e.g., CHADS _2_ VA _2_ Sc score = 7–9, recent [within 3 months] VTE) in whom heparin bridging would be considered with in-person management.

## Discussion

In this review of 4,609 patients assessed in a perioperative anticoagulation (heparin bridging) clinic over a 5-year period, we found that 80% of patients had the potential to be managed with a virtual care model and 7% would be likely to require an in-person model of care. The remaining 13% of patients could be managed with either in-person or virtual models. Taken together, our study suggests that virtual care has the potential to be an alternative to an in-person care model for perioperative anticoagulant management.

Virtual perioperative management of anticoagulation is feasible and potentially applicable to everyday clinical practice based on three key considerations. First, there is the availability of standardized, evidence-informed perioperative management protocols for patients who are receiving a DOAC or a VKA 15 ; these protocols are based on the practice-defining Perioperative Anticogulation Use for Surgery Evaluation (PAUSE), Bridging Anticoagulation in Patients who Require Temporary Interruption of Warfarin Therapy for an Elective Invaseive Procedure or Surgery (BRIDGE), and Perioperative Low-molecular-weight Heparin Bridging Treatment in Patients at High Risk for Arterial Thromboembolism (PERIOP-2) trials. 5 7 8 These simple, easy-to-use protocols are designed so that a wide array of clinicians could adopt them to patient care, ranging from a specialized anticoagulation clinic setting (such as that described herein) to an office-based primary care or family physician setting; moreover, standardized management protocols are preferred by patients who desire clear and consistent recommendations from the perioperative health care team. 16 Second, these protocols are easy to access and apply at the point of care as they are incorporated into web-based educational resources that are cost free (e.g., www.thrombosiscanada.ca ; http://mappp.ipro.org ; http://www.maqi2.org ) or subscription based (e.g., UpToDate: Perioperative Management of Patients Receiving Anticoagulants). Third, clinicians and patients are becoming increasingly attuned and receptive toward a virtual model of patient care, including the perioperative management setting. 12 13 Taken together, a virtual care model for perioperative anticoagulant management has the potential to simplify and increase accessibility of care for many patients, including those who live in remote communities and patients who have reduced mobility or limited access to transportation. 11 17 18 It can also improve access to care for people for whom time away from work, resulting in lost wages, and costs of travel and parking for in-person visits pose financial challenges.

In terms of key secondary findings, 5% of patients were identified as those in whom an in-person model was considered more appropriate, specifically to teach self-administration for perioperative low-molecular-weight heparin (LMWH) bridging in patients with an MHV. However, such care also has the potential to be delivered virtually with the availability of web-based educational videos or pharmacists that can instruct patients how to perform self-injections of LMWH. 10 There were 13% of patients with AF or VTE who were receiving either a DOAC or warfarin and were undergoing a high-bleed-risk surgery/procedure in whom, depending on individual patient factors, in-person or virtual management would be appropriate. For example, a DOAC-treated patient who was having a high-bleed-risk colon resection could be managed virtually, whereas a warfarin-treated patient within variable international normalized ratio (INR) control/warfarin compliance having the same surgery might require an in-person assessment. In addition, there may have been other patients with AF or VTE who were receiving warfarin who might have been considered for heparin bridging and in-person management. We were unable to reliably identify such patients, specifically at high risk of thromboembolism (e.g., CHADS _2_ VA _2_ Sc score = 7–9, recent [within 3 months] VTE = 7–9, VTE within the past 3 months). However, it is likely that such patients were infrequent; such high-risk patients comprise only 5 to 10% of all patients with AF or VTE. Moreover, the prevalence of such patients in future is likely to decrease as use of warfarin for AF or VTE is declining rapidly.

We acknowledge that a virtual model of perioperative anticoagulation care delivery may not be appropriate for some patients. This includes patients with hearing or visual impairment, those whose mother tongue is different than that used in such a clinic, and patients for whom there is no available family member or friend to assist with communication of perioperative management instructions. In addition, it may be impracticable to set up a virtual, video-based assessment for some patients, although the typical referral interval (10–14 days) should allow time to address such technical issues. Finally, there may some patients whose preference is an in-person model of care in this setting.

There are potential limitations of this study. First, it carries all potential limitations of a retrospective chart review, including the potential misclassification of patient-related data. However, the data captured in this study, specifically the clinical indication for anticoagulation, the anticoagulant used, and the type of surgery/procedure patients underwent, are objective datapoints that are less likely to be misrepresented, especially in a specialized anticoagulation clinic from which they were derived. Second, this was a single-center study in Ontario, Canada, and the patient population studied may not be representative of other clinical settings or in other jurisdictions. It is possible that a virtual model of care would be less applicable, for example, in clinics that assess predominantly patients with an MHV in whom heparin bridging may be more widely used. However, the makeup of patients in this clinic appears representative in terms of clinical indications for anticoagulation (i.e., AF > VTE > MHV) as other anticoagulation clinic settings. 19 20 Moreover, the surgeries/procedures patients underwent, with the majority being low-/moderate-bleed-risk surgeries/procedures, are consistent with that observed in studies involving unselected patients who are receiving a DOAC or VKA. 5 7 8 Third, we may have underestimated the proportion of patients who require heparin bridging as this was limited to warfarin-treated patients with an MHV and we did not include warfarin-treated patients with AF or VTE. Although our practice is, in general, not to bridge such patients with AF or VTE, we acknowledge this practice may differ in other clinical centers. Fourth, we acknowledge that our classification of patients according to bleeding risk is empiric and may be influenced by other patient-specific factors such as prior history of perioperative bleeding. 21 Finally, we acknowledge that there may be variability in how patients are managed, especially as to the need for perioperative heparin bridging, and this may affect estimates of patients in whom in-person management may be warranted. 21

In summary, this 5-year review of patients who were assessed in a perioperative (bridging) clinic identified a high proportion of patients in whom there is the potential for a virtual care model to be applied. Additional research is needed to support the safety of this approach, especially in patients at high risk of perioperative thromboembolism and bleeding.
